# PACS1-Neurodevelopmental disorder: clinical features and trial readiness

**DOI:** 10.1186/s13023-021-02001-1

**Published:** 2021-09-13

**Authors:** Abigail Van Nuland, Taruna Reddy, Farhad Quassem, Jean-Dominique Vassalli, Anne T. Berg

**Affiliations:** 1 Division of Neurology, Epilepsy Center, Ann & Robert H. Lurie Children’s Hospital of Chicago, 225 East Chicago Ave, Box 29, Chicago, IL 60611-2605 USA; 2 PACS1 Syndrome Research Foundation, 36 West Way, Old Greenwich, CT 06870 USA; 3 Faculty of Medicine, University of Geneva, Geneva, Switzerland; 4 Departments of Pediatrics and Neurological Surgery, Northwestern-Feinberg School of Medicine, Chicago, IL USA

**Keywords:** *PACS1*, Epilepsy, Neurodevelopment, Autism, Precision medicine, Trial readiness, Schuurs-Hoeijmakers syndrome

## Abstract

**Background:**

*PACS1*-Neurodevelopmental Disorder (*PACS1*-NDD) is an ultra-rare condition due to a recurrent mutation in the *PACS1* gene. Little systematically collected data exist about the functional abilities and neurodevelopmental morbidities in children with *PACS1*-NDD

**Methods:**

Parents of individuals with *PACS1-*NDD completed an on-line survey designed collaboratively by researchers, parents, and clinicians. Analyses focused on those with a confirmed R203W variant.

**Results:**

Of 35 individuals with confirmed variants, 18 (51%) were female. The median age was 8 years (interquartile range 4.5–15). Seventeen (49%) had a diagnosis of epilepsy. Twelve (40%, of 30 responding to the question) reported autism and (N = 11/30, 37%) reported features of autism. Most children walked independently (N = 29/32, 91%), had a pincer grasp (N = 23/32, 72%), could feed themselves independently (N = 15/32, 47%), and used speech (N = 23/32, 72%). Sixteen of twenty-nine (55%) had simple pre-academic skills. Neither epilepsy nor autism was associated with functional abilities or other clinical features (all *P* > 0.05).

**Conclusions:**

*PACS1*-NDD is a moderately-severe intellectual disability syndrome in which seizures occur but are not a defining or primary feature. Successful precision medicine clinical trials for this ultra-rare disorder must target important core features of this disorder and utilize assessment tools commensurate with the level of function in this clinical population.

## Introduction

*PACS1* neurodevelopmental disorder (*PACS1*-NDD) is a rare condition characterized by developmental delay, intellectual disability, dysmorphic features, and sometimes seizures [1–3]. *PACS1*-NDD was first identified when two individuals with similar dysmorphic features, and intellectual disability presented with the same de novo mutation in the *PACS1* gene [2]. This groundbreaking identification of Schuurs-Hoeijmakers Syndrome (*PACS1* disorder), has led to the description in the scientific literature of about 30 individuals [1–4], nearly all of whom have the same recurrent, de novo mutation in the *PACS1* gene, c.607C > T, with resulting protein change, p.R203W. There is one other pathogenic mutation reported in the *PACS1* gene, R203Q (p.608G > A), in one patient; it is the same amino acid that is changed, resulting, as for the R203W mutation, in the loss of a positive charge [5, 6]. This homogeneity in genotype makes *PACS1*-NDD an attractive potential target for precision medicine therapies, including gene-targeted therapies [1]. The available evidence suggests that the mutant protein affects cellular physiology, in a gain of function manner, through a toxic effect that results in a perturbation of the distribution and structure of organelles, and possibly of other functions. It might be possible to prevent the dominant negative “toxic” phenotype characteristic of *PACS1* syndrome, which is presumably due to the mutated protein, by inhibiting its synthesis (antisense nucleotides) or its effect (small molecules). Although it appears that patient-derived neural cells manifest such alterations, a link to the clinical features in patients with *PACS1*-NDD remains to be investigated.

Little is known about the specific impairments and relative strengths in *PACS1*-affected individuals that could provide insight into selection of one or more appropriate clinical assessment measures for use as an outcome in a randomized trial. Available descriptions of the condition highlight global developmental delay, and severe impairment but without providing further details [2, 7]. The Food and Drug Administration (FDA) has recently emphasized the importance of natural history data and of identifying the full range of disease manifestations for determining outcomes in clinical trials and of identifying valid measurements that are responsive to meaningful change over time [8]. To address this gap in knowledge, and in preparation for future clinical trials, a team of researchers, parents, and clinicians collaborated to identify domains and outcomes important to the families of children affected by *PACS1*-NDD and to implement a survey to provide a systematic characterization, based on parent-report, of the abilities and levels of function of young people with *PACS1*-NDD.

## Methods

Based on an initial survey of parents conducted by the *PACS1* Syndrome Research Foundation, areas of greatest concern for patients with *PACS1*-disorder were identified. We then adapted and augmented a series of parent-reported interview forms already utilized in over 200 families of children with developmental epilepsies and encephalopathies (DEE) [9]. Content included a medical checklist, seizure history, functional abilities, early academic skills, self-care, and therapies. For parent-reported outcomes of functional abilities, our framework reflected that commonly adopted in the rehabilitation setting—mobility, hand use, communication, eating [10] and relied on instruments that have been validated for parent report and widely used in the developmental literature.

### Functional outcome measures

#### Gross motor

The Gillette Functional Activity Level is a single 10-point question that ranges from “cannot take steps at all” to “walks, runs, climbs on level and uneven terrain without difficulty or assistance.” Those who were assessed at a level of 7 or higher (“Walks outside the home for community distances but only on level surfaces”) were also assessed with the Functional Activity Questionnaire, a 22-item instrument that assesses mobility skills ranging from “walk up and down stairs using a railing” (easiest) to “ice skates or roller skates” (hardest). Response choices were “easy, a little hard, very hard, and cannot do.” We added options for “too young,” which was scored as “cannot do: and “no opportunity,” which was scored as “not applicable.”

#### Communication

The Communication Function Classification System (CFCS) was used to measure communication on a five-point scale from “seldom communicates even with familiar persons”, to “communicates effectively with familiar and unfamiliar people” [10]. Parents were asked to indicate their child’s primary mode and other modes of communication (speech, sign language, gestures, sounds, communication device, eye gaze, or other) as well as number of words understood and used (0–5, 5–20, 20–100, > 100) and ability to combine 2 or 3-or-more words into short phrases and sentences.

#### Fine motor

Fine motor function was documented by parent-reported hand grasp (none, palmer, pincer), and ability to manipulate objects purposefully. We also administered items from the CDC developmental checklist relevant to hand use [11].

#### Eating

**Fig. 1:**
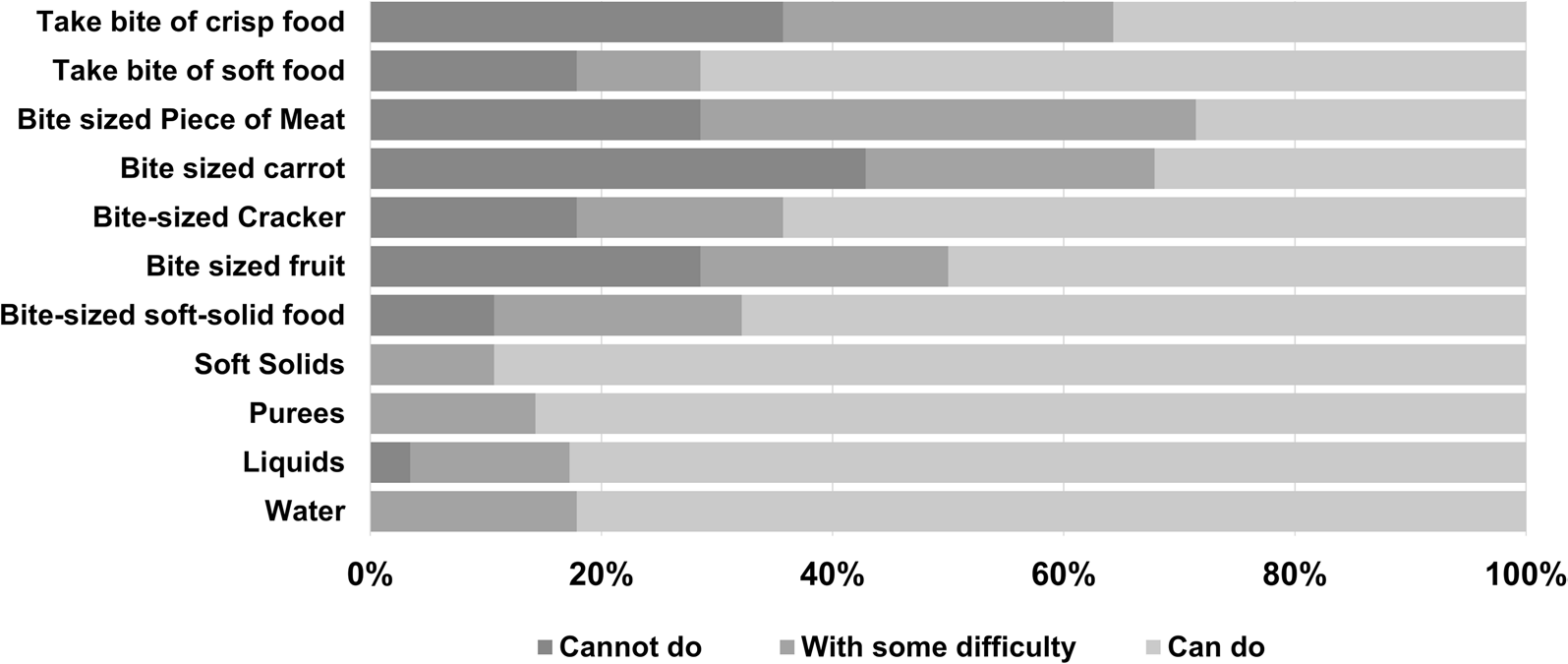
Responses to the Gillette functional activities questionnaire. (NA = not answered), *3 Removed for having FAQ1 score < 7. **Responses that indicate a child was too young for an activity were recorded as "cannot do." ^Responses recorded as N/A for reporting that child does not have the opportunity to complete this task

Parents were asked about the current use of a gastrotomy tube (G-tube) and whether feeding was partially or exclusively via tube. If feeding was not exclusively via tube, parents completed the Eating and Drinking Ability Classification System EDACS [10] to assess eating ability and safety. The EDACS is a five-point scale that ranges from “Independently eats and drinks safely and efficiently, no different from peers” to “Unable to eat or drink safely; tube feeding will or may be needed in the future.” We also asked a 5-point question developed for this study about independence for self-feeding that ranged from “child feeds self independently” to “Child cannot feed self, is dependent on someone else.” We also incorporated a series of questions about difficulty eating and swallowing liquids and foods posing varying levels of challenge ranging from water to taking a bite out of a crisp fruit or vegetable. Answer choices ranged from easy to cannot do (Fig. 1).

#### Self-care and activities of daily living

Toilet training was assessed as completely dependent, partially dependent, completely independent of others for assistance for children aged 2 years and older. Common activities of daily living related to self-care (e.g. dressing and using clothing fasteners) were also assessed.

#### Elementary academic skills

Parents of children 3 years and older were asked if their child had any pre-academic skills (e.g. letter recognition, scribbling). If they answered in the affirmative, a checklist of 17 academic skills was queried.

#### Medical

Parents and collaborators provided lists of medical concerns common in *PACS1* disorder. Standard checklists based on those used in clinical care for review of systems were constructed and included pregnancy, labor and delivery, gastrointestinal, cardiovascular, dermatologic, pulmonary, endocrine, dental, orthopedic, infections, muscular, allergies, autonomic, hearing, and vision. Parents were provided definitions and descriptions of specific seizures types to assist with the seizure history questions [12].

The questionnaires were administered in CLRINIX, a web-based software designed to facilitate multi-site clinical research [13]. Portions of the questionnaires used in the analyses for this presentation are included as an appendix.

### Participants and data collection

Participants were recruited through an announcement disseminated through the *PACS1* family group (September 2019–May 2020). The questionnaire was available in English only. To be included in these analyses, the parents were required to provide a copy of the child’s genetic testing report confirming the c.607C > T (p.R203W) variant. Data were analyzed in SAS 9.4 and R-Studio 1.2. Methods of analysis included χ^2^ tests, and non-parametric tests as appropriate for the data. All study procedures were approved by the Institutional Review Board at Lurie Children’s Hospital. Informed consent and GDPR consent, when required, were obtained electronically through the CLIRINX website.

## Results

### Demographics

Thirty-five individuals with a confirmed *PACS1* R203W mutation participated in this study. Eighteen (51%) were female, and the median age at the time of the survey was 8 years (IQR 4.5–15 years; max = 26 years). Participants came from the United States (N = 13), United Kingdom (N = 5), Spain (N = 4), Australia (N = 3), Canada (N = 3), Netherlands (N = 2), and one from each of five other countries.

### Initial presentation

The median age at initial evaluation for concerns was 5 months, and 90% were evaluated by 21 months of age. The most common reasons for first bringing a child to medical attention were delayed development (N = 18, 51%) and seizures (N = 10, 29%). Other individual reasons included dysmorphic features (N = 2, 6%), bilateral coloboma (N = 1, 3%), tachypnea (N = 1, 3%), breath holding episodes (N = 1, 3%), and difficulty gaining weight (N = 1, 3%). The median age at *PACS1* molecular diagnosis was 5 years, and 90% were diagnosed by 14.5 years.

Seventeen (49%) had an epilepsy diagnosis, 15 (43%) never had epilepsy, and for three (9%), the parents were unsure. The median age at first seizure of any kind was 12 months (IQR 2 Weeks to 24 months; max = 18 years). Two parents reported the first seizure was accompanied by fever and one by vaccinations. The most common seizure types reported by parents were generalized convulsions (N = 6/17, 35%), infantile spasms (N = 3/17, 18%), absence (N = 2/17, 12%), myoclonic (N = 2/17, 12%), focal motor seizures (N = 2/17, 12%), clonic (N = 1/17, 6%), tonic (N = 1/17, 6%) and hemiconvulsions (N = 1/17, 6%). Only two children (ages 4.5 and 12.5 years) were reported to have had seizures within the six months prior to the survey; one had weekly and the other monthly seizures. For children with more remote seizures, the median age at last seizure was 4.4 years (IQR 2.9–6.3 years; max = 18). Two participants were unsure of the age at last seizure.

At least one EEG was performed in 29 (83%) children including all who had an epilepsy diagnosis and 10 (56%) of those without an epilepsy diagnosis. Studies included brief routine EEGs without sleep (N = 21/29, 72%), overnight with sleep (N = 9/29, 31%), and routine brief EEG with sleep (N = 8/29, 27%). Electrographic status epilepticus in sleep (ESES) was reported in 4 (11%) participants. A brain MRI had been performed in 28 (80%) children. Parent-reported results were abnormal (N = 13, 37%) or equivocal (N = 9, 26%). Specific abnormalities were not elicited.

### Behavior

Twelve out of thirty (40%) children carried an autism diagnosis and another 11/30 (37%) were reported to have features of autism without a formal diagnosis. Attention deficit hyperactivity disorder (N = 4/30, 13%), oppositional defiant disorder (N = 3/30, 10%), obsessive compulsive disorder (N = 2/30, 7%), anxiety (N = 2/30, 7%), and aggression (N = 1/30, 3%) were formally diagnosed in some children. Twelve participants had used applied behavior analysis (“ABA”) therapy, and 8/12 (67%) rated the therapy as having a good to excellent effect. Nine (82%) of 11 children who had used sensory integration therapy reported good to excellent effect.

There was no association between a diagnosis of epilepsy and a diagnosis of autism or of having autistic features. Of 28 for whom information about epilepsy and autism was reported, epilepsy was reported in 4/28 (14%) of children without autism, 7/28 (25%) of children with a diagnosis of autism, and 3/28 (11%) of children with features of autism.

### Medical history

Half of children were reported to have had neonatal feeding difficulty (N = 18, 51%). Other conditions in the neonatal period that were reported included neonatal seizures (N = 6, 17%), jaundice (N = 6, 17%), and failure to thrive (FTT) (N = 5, 14%). Eight (23%) children required care in the neonatal intensive care unit (NICU).

Abnormalities in muscle tone and movement were the most common types of concerns endorsed (Table 1) and included hypotonia (N = 15, 43%), hypertonia (N = 6, 17%), and ataxia (N = 3, 9%). Other commonly endorsed concerns were gastrointestinal disorders, specifically constipation (N = 14, 40%), dental (N = 14, 40%), and orthopedic such as scoliosis and kyphosis (N = 10, 29%). Other types of disorders were elicited at levels that might be typical in the general population with the use of a similar checklist.

**Table 1:** Medical morbidities reported in *PACS1* children

System	N (%) [N current]^a^	Specific findings
Muscle tone, movement	22 (63%) [20]	Hypotonia (N = 15), Hypertonia (N = 6), Ataxia (N = 3), Myoclonus (N = 1), Dystonia (N = 1), Tremor (N = 1)
Gastrointestinal	14 (40%)	Constipation (N = 14), GERD (N = 1)
Cardiac	12 (34%) [4]	Ventricular septal defect (N = 4), Atrial septal defect (N = 4), Patent ductus arteriosus (N = 3), Single ventricular defect (N = 1)
Skin conditions	5 (14%) [4]	Excessive moles (N = 3), Atopic dermatitis (N = 1), Acne (N = 1), Portwine stain birthmark (N = 1)
Lungs	4 (11%) [1]	Asthma (N = 1), Reactive airway disease (N = 1), Chronic bronchitis (N = 1)
Endocrine	9 (26%) [8]	Short stature (N = 3), delayed puberty (N = 2), hypothyroidism (N = 2), precocious puberty (N = 1)
Dental	14 (40%)	Thumb sucking (N = 5), bruxism (N = 5), late baby tooth eruption (N = 5), late permanent teeth (N = 5), cavities (N = 3), broken teeth (N = 1)
Infections	4 (11%) [1]	Common cold (N = 3), gastrointestinal illnesses (N = 1), skin wounds get easily infected (N = 1)
Orthopedic	10 (29%)	Scoliosis (N = 4), Kyphosis (N = 2)
Allergy	8 (23%) [6]	Certain foods (N = 4), pets (N = 1), pollen (N = 1)

^a^Number of individuals who have experienced this medical morbidity (%) [Number of individuals currently experiencing this medical morbidity]

Other concerns included difficulty judging distances (N = 9, 26%), and impaired depth perception (N = 7, 20%). Balance and steadiness problems (N = 24, 69%) were common, and were attributed by parents to poor muscle tone (N = 17, 49%) and to visual-motor integration difficulties (N = 16, 46%).

### Basic functional abilities

**Fig. 2:**
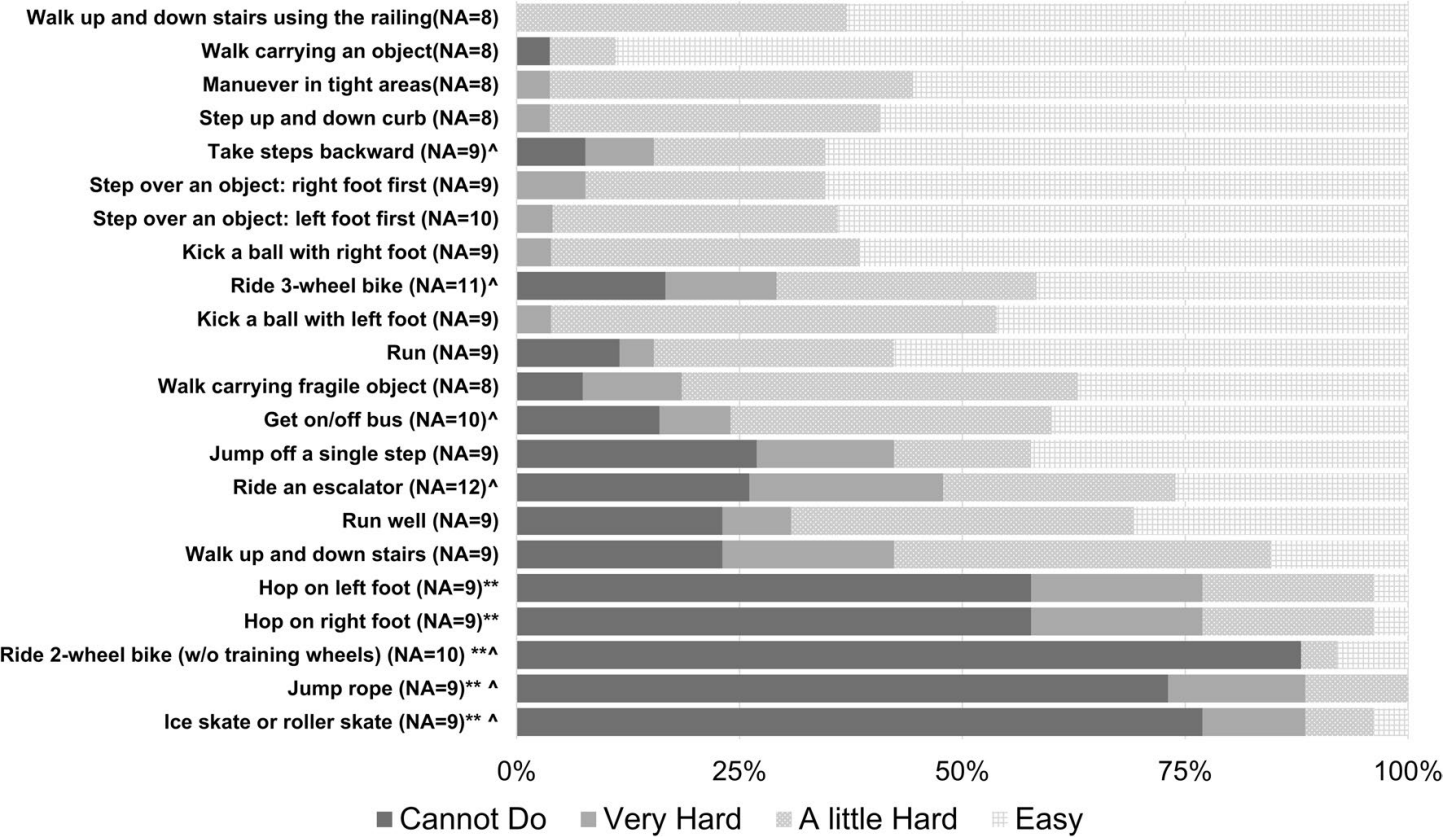
Ability to eat or drink foods that pose increasing challenge

For children ≥ 2 years-old (N = 34), the median FAQ-level was 8.5 (IQR 8–9.8), corresponding to "Walks outside the home for community distances, but usually requires minimal assistance or supervision for safety" and "Walks outside the home for community distances, easily gets around on level ground, and uneven terrain, but has difficulty or requires minimal assistance with running, climbing, or stairs.” Three children scored < 7 including 2 who couldn’t walk and 1 who could walk short distances (15–20 feet) outside but used a medical stroller for community distances. Of 29 children for whom the FAQ was completed (scored ≥ 7 on the FAQ1), the FAQ-22 responses indicated that children could, without difficulty, walk up and down stairs with a railing (100%) and walk while holding an object in their hands (96%). Few children, however, could do more complex skills such as jump rope (11%) or hop (22%) (Fig. 2).

#### Hand use

Of 32 participants with hand-use information, all participants were able to grasp and manipulate objects with their hands; most (N = 27, 84%) had a pincer grasp. Seventeen (53%) were reported to favor their right hand, 6 (19%) favored their left, and 3 (9%) had no clear hand preference. Most had acquired fine motor skills appropriate for a 2 year-old (manipulate clay N = 20, 63%, complete a three-piece puzzle, N = 16, 50%, and use a spoon correctly N = 19, 59%), but fewer had the fine motor skills of 3 and 4 year old (e.g. touch each finger to thumb N = 9, 28%, and color a picture within the lines N = 6, 19%).

#### Communication

Of the 32 who provided information on communication abilities, 23 (72%) had spoken speech, and 21 (66%) used speech as the primary mode of communication. Others primarily relied on gestures (N = 4, 13%), sign language (N = 1, 3%), sounds (N = 1, 3%), and eye pointing (N = 1, 3%). Of those with speech, 19 (59%) combined three or more words into sentences and, 18 (56%) had an expressive vocabulary of > 100 words. Only 11 (34%) spoke clearly (“anyone can understand” or “some words or phrases may need to be repeated”).

#### Eating ability and safety

Of the 29 children whose parents completed information on eating, none had a G-tube. On the EDACS, 10 of the 29 (34%) were rated as eating no differently than peers in terms of need for supervision and ability to handle food of different textures, 7 (24%) were safe while eating but needed additional time and had difficulty with higher volumes of food, 10 (34%) had limitations and primarily eating soft and pureed foods, and 1 (3%) was unable to eat or swallow without risk of aspiration.

Most children could drink water (N = 23, 82%), other liquids (N = 24, 86%), and purees (N = 24, 86%) and could eat bite-sized solid soft food (N = 19, 68%) without any difficulty or safety concerns (Fig. 1). Fewer could eat a bite-sized piece of meat (N = 8, 29%) or take a bite out of a crisp piece of food (e.g. apple) (N = 10, 36%).

#### Academics

Half of children ≥ 3 years-old (N = 16/29, 55%) reported pre-academic skills such as counting to 10 (N = 13) and writing own name (N = 6). There was a moderate correlation between number of academic skills obtained and age (r = 0.40, *P* = 0.02).

#### Self-care

Of 29 children aged ≥ 3-years-old, 10 (34%) were completely dependent on caregivers for toileting needs, 11 (38%) were partially toilet trained, and 7 (24%) were completely independent for toileting needs. Few could dress themselves (N = 5, 17%), use a zipper (N = 12, 41%), and fasten and unfasten large buttons (N = 6, 21%).

#### Eating independence

Data were available for 29 children of whom 15 (52%) fed themselves independently 8 (28%) with some assistance, 2(7%) with considerable assistance, and 4 (14%) were completely dependent on someone else. Seventeen (59%) could use a fork, and 22 (76%) used a spoon to feed themselves. Only one child could cut food.

#### Sleep

Of the 30 parents reporting on sleep, 27 (90%) indicated that their children slept 8–11 h a night. No participants reported nocturnal seizures. Most children usually slept through the night; however, 11 (37%) had night-time awakenings that occurred at least once per week, and 5 (17%) reported night-time awakenings less than weekly.

#### Age, epilepsy and function

We found few associations between any of these outcomes and the history of epilepsy or autism (Table 2). Apart from epilepsy diagnosis, none of the abilities or outcomes described above was strongly associated with age at the time of the survey (Table 3).

**Table 2:** Functional abilities in *PACS1*-NDD by epilepsy diagnosis, and autism diagnosis

	Total (N = 35)	Epilepsy diagnosis (N = 17)	No epilepsy diagnosis (N = 15)^a^	*P* value^b^	Autism diagnosis (N = 12)	Autism features (N = 11)	No diagnosis (N = 8)^c^	*P* value^b^
*Communication*								
Effectively communicates with everyone	1 (3%)	0 (0%)	1 (7%)	0.27	0 (0%)	0 (0%)	1 (13%)	0.81
Effectively communicates with adults	11 (31%)	4 (24%)	6 (40%)		6 (50%)	2 (18%)	2 (25%)	
Effectively communicates with familiar people	7 (20%)	3 (18%)	3 (20%)		2 (17%)	3 (27%)	1 (13%)	
Inconsistently communicates	7 (20%)	4 (24%)	2 (13%)		2 (17%)	3 (27%)	2 (25%)	
Seldom communicates	2 (6%)	1 (6%)	1 (7%)		1 (8%)	0 (0%)	1 (13%)	
*Fine motor*								
Holds large objects	4 (11%)	0 (0%)	4 (27%)	0.03	1 (8%)	1 (9%)	1 (13%)	1
Pincer grasp	27 (77%)	14 (82%)	10 (67%)		10 (83%)	9 (82%)	6 (75%)	
*Gross motor*								
Median score (IQR, max = 10)	8.5 (8–9.8)	8 (7.25–9)	9 (8–10)	0.56	9 (7.5–9.5)	9 (8–9.8)	8 (7.5–9.5)	0.30
*Academic skills*								
Yes	16 (46%)	6 (35%)	7 (47%)	1.00	7 (58%)	6 (55%)	3 (38%)	0.52
No	13 (37%)	6 (35%)	7 (47%)		4 (33%)	2 (18%)	4 (50%)	
*Toileting*								
Independent	10 (29%)	3 (18%)	6 (40%)	0.12	2 (17%)	2 (18%)	3 (38%)	0.86
Partially dependent	11 (31%)	5 (29%)	6 (40%)		6 (50%)	1 (9%)	4 (50%)	
Completely dependent	7 (20%)	4 (24%)	1 (7%)		2 (17%)	3 (27%)	3 (38%)	
*Eating*								
Independent	15 (43%)	6 (35%)	8 (53%)	0.69	6 (50%)	4 (36%)	4 (50%)	0.91
Some assistance	8 (23%)	4 (24%)	2 (13%)		3 (25%)	3 (27%)	1 (13%)	
Considerable assistance	2 (6%)	1 (6%)	1 (7%)		1 (8%)	0 (0%)	1 (13%)	
Dependent	4 (11%)	1 (6%)	3 (20%)		1 (8%)	1 (9%)	1 (13%)	
*Nocturnal awakenings*								
6–7 nights/week	1 (3%)	0 (0%)	0 (0%)	0.21	0 (0%)	1 (9%)	0 (0%)	0.97
3–5 nights/week	3 (9%)	2 (12%)	1 (7%)		3 (25%)	0 (0%)	0 (0%)	
1–2 nights/week	7 (20%)	4 (24%)	2 (13%)		1 (8%)	3 (27%)	2 (25%)	
< 1 night/week	5 (14%)	2 (12%)	3 (20%)		3 (25%)	0 (0%)	2 (25%)	
Never	10 (29%)	3 (18%)	5 (33%)		3 (25%)	2 (18%)	4 (50%)	

^a^Does not include 3 who were unsure of epilepsy diagnosis

^b^Mantel–Haenszel χ^2^ for trend

^c^Does not include 1 who was unsure of autism diagnosis

**Table 3:** Functional abilities by median age

Level	Median age in years (IQR)	*P* value (DF)^a^
*Communication*		0.10 (4, 23)
Effectively communicates with everyone (N = 1, 4%)	3	
Effectively communicates with adults (N = 11, 39%)	8 (4.5–11, max = 12.5)	
Effectively communicates with familiar people (N = 7, 25%)	15.5 (7–16, max = 19)	
Inconsistently communicates (N = 7, 25%)	13 (9–15, max = 26)	
Seldom communicates (N = 2, 7%)	4 (3.5–5, max = 6)	
*Fine motor*		0.42 (1, 29)
Holds large objects (N = 4, 13%)	7 (2–12, max = 15.5)	
Pincer grasp (N = 27, 87%)	8 (5.5–14, max = 26)	
*Gross motor*		0.83^b^
Spearman correlation between age and FAQ score (N = 30)	r_s_ = -0.04	
*Academic skills*		0.09 (1, 27)
Yes (N = 16, 55%)	11 (7.5–13.7, max = 26)	
No (N = 13, 45%)	5.83 (3–8, max = 19)	
*Autism diagnosis*		0.63 (2, 28)
Diagnosis (N = 12, 39%)	9.5 (5.5–11.5, max = 16.5)	
Features (N = 11, 35%)	13 (6.5–15, max = 18.5)	
No diagnosis (N = 8, 26%)	5.5 (3–10.5, max = 26)	
*Epilepsy diagnosis*		0.05 (1, 30)
Yes (N = 17, 53%)	9 (7–16.5, max = 26)	
No (N = 15, 47%)	5 (3.5–11, max = 15.5)	
*Toileting*		0.24 (2, 25)
Independent (N = 7, 25%)	11.5 (8–14.5, max = 16.5)	
Partially dependent (N = 11, 39%)	11 (7–13, max = 26)	
Completely dependent (N = 10, 36%)	5 (4–11, max = 15.5)	
*Eating*		0.06 (3, 25)
Independent (N = 15, 52%)	11 (5.5–14.5, max = 26)	
Some assistance (N = 8, 28%)	12 (8–15, max = 19)	
Considerable assistance (N = 2, 7%)	5.5 (4–6.5, max = 8)	
Dependent (N = 4, 14%)	3.3 (2–4, max = 6)	
*Nocturnal awakenings*		0.46 (5, 24)
6–7 nights/week (N = 1, 4%)	15	
3–5 nights/week (N = 3, 12%)	8 (6.5–9.5, max = 11)	
1–2 nights/week (N = 7, 27%)	6 (4–9.5, max = 13)	
< 1 night/week (N = 5, 19%)	8 (3–11, max = 16.5)	
Never (N = 10, 39%)	11 (8–15.5, max = 26)	

^a^One way analysis of variance was used to obtain *P* values

^b^Spearman correlation

Children with *PACS1* disorder were seen by a median of 6 (IQR 4–9, max 15) different types of specialists other than neurologists. Occupational (N = 22/29, 76%) and speech (N = 21/29, 72%) therapists were the most common. Most participants (N = 18/29, 62%) reported having a therapy appointment at least 2 times per week. About a third of parents (N = 10/29, 34%) felt that care was not well-coordinated across specialists.

## Discussion

Our findings based on 35 children with documented *PACS1* R203W mutations largely support the initial description by Schuurs–Hoiejmakers [2] and a separate description of 16 patients by Seto [5] of *PACS1*-NDD as a moderately severe neurodevelopmental disorder. Presentation is early in life, primarily with developmental delay or seizures. Although most children had basic functional independence for walking, communicating, eating, and hand use, function in these domains was impaired as reflected by the assessments on the FAQ-22, eating abilities, hand use for feeding and dressing, and ability to eat certain foods. Communication was limited, and only about half had rudimentary academic skills.

Only half of children had a history of epilepsy, which is similar to the 63% (12/19) from the original report [1]. With one exception, seizures in our series began within the first two years of life and were mostly well-controlled. Generalized convulsions were the most common seizure type; however, other seizure types were endorsed. Although we provided definitions for the different seizure types, it is unclear whether the term “spasm” was used colloquially or if these children had actual epileptic spasms. We were able to review video of one child whose parents believed had epileptic spasms, and the event was of a prolonged, slow, rhythmic, focal nature but was not an epileptic spasm.

There was no association between the occurrence or severity of other morbidities in children and diagnosis of epilepsy or of autism or report of autistic features. This was somewhat unexpected as seizures presage poorer outcome in several settings including after acute injuries such as neonatal hypoxia [14] or head trauma [15] or in the setting of a chronic, degenerative condition such as Alzheimer or Parkinson disease or multiple sclerosis [16–18]. A similar lack of association was seen in of SCN2A-associated disorders; those with epilepsy were similar to those without with respect to several medical morbidities that were assessed [19]. Studies of children with Dravet syndrome [20] and KCNQ2-associated epilepsy [21] also found no clear correlation between seizure burden and cognitive measures. These types of findings from different disorders tend to emphasize the importance of the genotype itself above and beyond the effects of seizures themselves.

No children in our genotype-confirmed series had gastrotomy tubes; however, g-tube use was reported in two children whose parents did not provide genetic testing confirmation and who were thus excluded from this report. By contrast, four (21%) children in the original series of 19 patients, had g-tubes [1], and a subsequent study reported 1/8 (13%) to have a g-tube [4]. Even though all the children in our *PACS1*-confirmed series ate by mouth, the parent rating on the EDACS indicated that most children were impaired in eating relative to their peers and few could eat challenging foods well. Whether this is due to impaired strength or oromotor coordination would require clinical assessment.

Children with *PACS1*-NDD have a high reported prevalence of a large number of medical morbidities. This high burden of medical morbidity is seen in many, perhaps all neurodevelopmental disorders [22]. To emphasize the magnitude of this burden, we compared the frequencies reported in our series to those reported in a recent population-based study of children in [23]. The *PACS1*-NDD series had a much higher prevalence of several medical conditions including musculoskeletal disorders (population 4% vs *PACS1*-NDD: 62%), gastrointestinal disorders (population: 5% vs *PACS1*-NDD: 41%), cardiac disorders (population: < 1% vs *PACS1*-NDD: 32%), and endocrine disorders(population 1% vs *PACS1*-NDD: 26%) [23]. While there are difficulties in comparing documented diagnosis in the medical record (population report) with parent reported diagnoses, these tenfold or greater differences suggest a substantially elevated risk of these other medical conditions in children with *PACS1*-NDD, which deserves more detailed characterization.

Our study had significant limitations. First, all data were parent-reported, and not derived from clinical evaluations or medical records. On the other hand, the data were systematically collected, and many of the instruments we used such as the FAQ [24], FMS [25], CFCS [26], and EDACS [27] have been validated for use with parent report. Further, we did require a copy of the child’s genetic test report to confirm that each child had the specific recurrent variant associated with *PACS1*-NDD. Comparison of children with (N = 35) and without (N = 22) variant confirmation suggested little or no differences between the groups.

*PACS1*-NDD is currently an ultra-rare disorder. The authors of a recent literature review referred to 36 cases identified in the literature [5]. We have no way of determining how many of the children in our series are new or whether they have been reported in other series; however, we are aware of at least two from the original report [1] who also participated in this study.

A key motivation for this survey was to delineate the range of possible clinical domains that might be used as outcomes in a future clinical trial. Seizures are a common trial outcome; however, a large proportion of children with *PACS1*-NDD do not have seizures, and most of those who have a history of epilepsy have well-controlled seizures. Consequently, seizure may not be an efficient trial outcome, especially considering how rare *PACS1*-NDD is. The FDA has provided a guidance to industry for design of randomized trials for rare diseases. This guidance emphasizes that trials need to target outcomes of importance to patients, those that are life-limiting and life altering [8], trials should utilize outcome measures that are relevant to the patient population’s condition and sensitive to meaningful change.

For a future randomized trial of this exceedingly rare disorder, it will be essential to identify or develop clinical outcome assessment measures that represent common, key aspects of *PACS1*-NDD that might be reasonably expected to change with therapy.

Rather than a form of epilepsy or epileptic encephalopathy, *PACS1*-NDD appears to be better described as an intellectual disability syndrome, perhaps akin to Down syndrome in terms of severity in intellectual and functional impairment. There is also a prominent autism component which needs further definition as autism diagnosis is challenging in the presence of intellectual disability, and autistic features can be nonspecific symptoms in a child with intellectual disability [28]. Our series also identified motor disturbances in gait (balance and coordination) and eating (due to tone or impaired oral-motor coordination).

Appropriate measures, including parent-reported outcomes, performance measures, and biomarkers that assess these domains in this population will be needed if these become targeted outcomes for randomized trials. Such measures will need to be sufficiently granular and sensitive to change commensurate with the levels of abilities and impairments seen in children with *PACS1*-NDD. This is similar to the path taken by Angelman investigators [29, 30]. Our survey represents a baseline from which to start considering these issues by providing systematically collected data about the level of function and the types of conditions most important to parents of children with *PACS1*-NDD.

## Data Availability

The datasets generated and/or analyzed during the current study are not publicly available due to containing potentially identifying information but are available from the corresponding author on reasonable request.
